# Impact of Positive Peritoneal Cytology on Prognosis in Stage IA Endometrial Cancer: A Retrospective Cohort Study in the Era of Minimally Invasive Surgery

**DOI:** 10.7759/cureus.82469

**Published:** 2025-04-17

**Authors:** Shinichi Togami, Nozomi Fufuzono, Mika Fukuda, Hiroaki Kobayashi

**Affiliations:** 1 Department of Obstetrics and Gynecology, Faculty of Medicine, Kagoshima University, Kagoshima, JPN

**Keywords:** endometrial cancer, peritoneal cytology, prognosis, recurrence, survival

## Abstract

Objective: The prognostic significance of positive peritoneal cytology (PC) in early-stage endometrial cancer remains debated, particularly following its exclusion from the 2008 International Federation of Gynecology and Obstetrics (FIGO) staging system. This study aimed to evaluate the impact of positive PC on recurrence and survival outcomes in patients with stage IA endometrial cancer, considering contemporary surgical practices, including minimally invasive surgery.

Methods: This retrospective cohort study included 325 patients diagnosed with stage IA endometrial cancer who underwent primary surgical treatment at Kagoshima University Hospital between 2010 and 2022. PC was assessed intraoperatively, and patients were stratified into positive and negative cytology groups. Clinicopathological characteristics, recurrence-free survival (RFS), and overall survival (OS) were compared using Kaplan‒Meier analysis. Cox proportional hazards regression was performed to identify independent prognostic factors.

Results: Positive PC was identified in 5.8% (19/325) of cases. Patients with positive cytology had significantly lower five-year RFS (72.7% vs. 93.7%, p = 0.0001) and OS (82.1% vs. 96.1%, p = 0.0001). Cox regression analysis identified positive PC as the strongest independent predictor of recurrence (HR 8.33, 95% CI: 2.41-25.8) and mortality (HR 14.2, 95% CI: 2.22-90.3). In addition, advanced age (≥60 years) was significantly associated with decreased survival.

Conclusion: Positive PC is a significant adverse prognostic factor in stage IA endometrial cancer, associated with increased recurrence and reduced survival. Future large-scale prospective studies are needed to determine its role in risk stratification and management of stage IA endometrial cancer.

## Introduction

Endometrial cancer is among the most common gynecologic malignancies, generally associated with a favorable prognosis and an estimated five-year survival rate of approximately 80% [1]. The standard treatment for endometrial cancer involves a simple total hysterectomy with bilateral salpingo-oophorectomy, and lymph node dissection or biopsy when indicated [2]. The staging of endometrial cancer is determined based on multiple pathological factors identified in the resected specimens, which subsequently guide the need for adjuvant therapy [3]. Consequently, each prognostic factor is crucial in determining patient outcomes.

In the 2008 International Federation of Gynecology and Obstetrics (FIGO) classification, peritoneal cytology (PC) was excluded from the staging criteria for endometrial cancer, and stage IIIA was thereafter limited to cases involving serosal or adnexal invasion, excluding PC as a factor in stage determination [4]. This exclusion was largely influenced by studies suggesting that positive PC does not significantly impact prognosis [5,6]. However, recent studies have reported a significant association between positive PC and reduced survival in patients with endometrial cancer [7-9]. Although PC is no longer included in the FIGO staging system, the National Comprehensive Cancer Network guidelines still recommend its evaluation during surgery, indicating its potential prognostic relevance [3].

The exclusion of positive PC from the staging criteria may have had the greatest impact on stage IA endometrial cancer (FIGO 2008) with positive cytology. Previously classified as stage IIIA and typically managed with adjuvant therapy, these cases are now considered low risk for recurrence and do not routinely undergo adjuvant treatment. In addition, the surgical approach to endometrial cancer has shifted from open surgery to minimally invasive surgery (MIS), including laparoscopic and robot-assisted approaches, since the 2008 FIGO classification update. Given these substantial changes, limited studies have evaluated the prognostic impact of positive PC in stage IA endometrial cancer (FIGO 2008), considering these recent advancements. This study aimed to analyze the prognostic impact of positive PC in stage IA endometrial cancer (FIGO 2008) and reassess its clinical significance.

## Materials and methods

Study population

This retrospective study included patients who underwent surgery at Kagoshima University Hospital between January 2010 and January 2022 and were pathologically diagnosed with stage IA endometrial cancer (FIGO 2008). Eligible cases included those who underwent at least a total hysterectomy and had histologically confirmed endometrial cancer with ≤ 50% myometrial invasion. The exclusion criteria included patients who received neoadjuvant chemotherapy, those with sarcomatous histology (excluding carcinosarcoma), those with synchronous malignancies, and those for whom PC was not performed intraoperatively. Clinical and pathological data, including patient age, body mass index (BMI), final histological subtype, lymphovascular space invasion (LVSI), surgical approach (open surgery vs. MIS), adjuvant therapy, recurrence, and survival outcomes, were extracted from electronic medical records. The study was approved by the Institutional Review Board of Kagoshima University Hospital (Approval No. 240153).

Clinical data analysis

Surgical staging was determined based on the 2008 FIGO classification. PC was considered positive if malignant cells were identified, whereas negative cytology included cases without malignant cells or with atypical cells of uncertain malignancy. Patients were stratified into two groups based on age (< 60 vs. ≥ 60 years) and BMI (< 30 vs. ≥ 30). Histological subtypes were categorized as endometrioid carcinoma (grades 1, 2, and 3), serous carcinoma, clear cell carcinoma, and other types. Surgical procedures included simple hysterectomy with bilateral salpingo-oophorectomy, with or without lymphadenectomy/biopsy. Surgical approaches were classified as open surgery or MIS (laparoscopic or robotic-assisted surgery). MIS was introduced in December 2016. In all MIS cases, the external cervical OS was sutured closed before surgery, and uterine manipulators were not used. PC was collected before uterine extraction in both open and MIS procedures. Adjuvant therapy was administered in cases of grade 3 endometrioid carcinoma, non-endometrioid histology, or LVSI. Recurrence was diagnosed based on clinical examination, cytological/histological analysis, tumor markers, and imaging studies. Recurrence-free survival (RFS) was defined as the interval from primary surgery to the first recurrence, and overall survival (OS) was defined as the time from surgery to the last follow-up or death from endometrial cancer.

Statistical analysis

Comparisons of clinicopathological characteristics between the positive and negative PC groups were performed using the chi-square test. Survival analysis was conducted using Kaplan‒Meier survival curves, with statistical significance assessed via the log-rank test. A p-value < 0.05 was considered statistically significant. Cox proportional hazards regression models were used to identify independent prognostic factors associated with recurrence and survival. Statistical analysis was performed using JMP software (version 14, SAS Institute Inc., Cary, NC, USA).

## Results

A total of 325 patients with stage IA endometrial cancer were analyzed, including 19 (5.8%) with positive PC and 306 (94.2%) with negative PC. The overall clinicopathological characteristics are summarized in Table 1. The two groups were generally similar in terms of age distribution and histological subtype. Although BMI differed significantly between groups, other factors such as LVSI, surgical approach (open vs. MIS), and the proportion of patients receiving adjuvant therapy did not show statistically significant differences.

**Table 1:** Clinicopathological characteristics of 325 patients with stage IA endometrial cancer in this study PC: peritoneal cytology, BMI: body mass index, LVSI: lymphovascular space invasion, MIS: minimally invasive surgery Statistical comparisons were conducted using chi-square (χ²) tests for categorical variables. A p-value < 0.05 was considered statistically significant.

Characteristic	Positive PC (n = 19)	Negative PC (n = 306)	Test Statistic	P-value
Age <60/≥60	15 (79.0%)/4 (21.0%)	182 (59.5%)/124 (40.5%)	χ² = 2.85	0.09
BMI <30/≥30	14 (73.7%)/5 (26.3%)	238 (77.8%)/68 (22.2%)	χ² = 15.24	<0.0001
Final pathology	Endometrioid 18 (94.7%), serous 1 (5.3%), others 0	Endometrioid 276 (90.2%), serous 13 (4.3%), clear cell 9, others 8	χ² = 0.92	0.77
LVSI (no/yes)	19 (100%)/0 (0%)	279 (91.2%)/27 (8.8%)	χ² = 1.80	0.18
Surgical procedure (open/MIS)	11 (57.9%)/8 (42.1%)	135 (44.1%)/171 (55.9%)	χ² = 1.38	0.24
Adjuvant therapy (no/yes)	13 (68.4%)/6 (31.6%)	249 (81.4%)/57 (18.6%)	χ² = 1.90	0.17
Recurrence (no/yes)	14 (73.7%)/5 (26.3%)	288 (94.1%)/18 (5.9%)	χ² = 11.33	0.0008

The recurrence rate was markedly higher in the positive PC group compared to the negative PC group, with peritoneal dissemination being the predominant site in the former. In contrast, recurrences in the negative group were more widely distributed, including the vaginal cuff, lymph nodes, and distant sites. Detailed recurrence patterns are presented in the text and summarized in Table 1.

**Figure 1:**
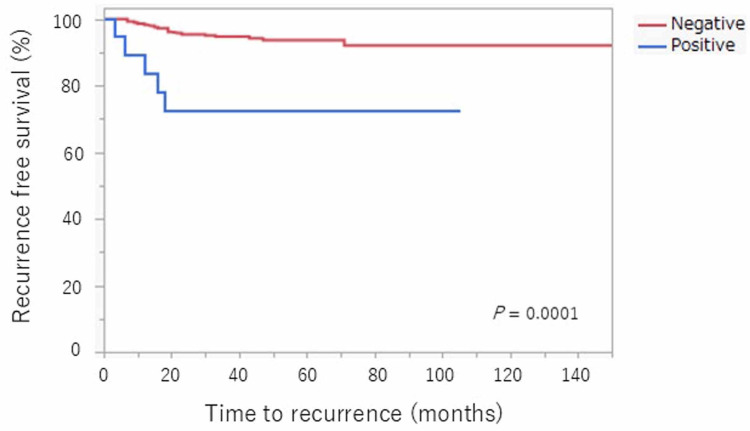
Five-year recurrence-free survival rate

**Figure 2:**
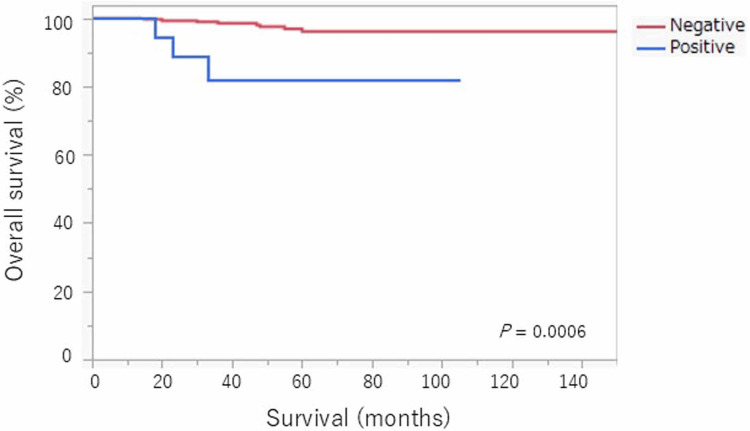
Five-year overall survival rate

Kaplan-Meier survival analysis demonstrated significantly reduced RFS and OS in the positive PC group, with five-year RFS and OS rates of 72.7% and 82.1%, respectively, compared to 93.7% and 96.1% in the negative PC group (p = 0.0001 for both; Figures 1, 2). These findings underscore the prognostic significance of positive cytology in stage IA endometrial cancer.

Table 2 presents the Cox proportional hazards regression analysis for recurrence and survival. Older age (≥ 60 years), open surgery, and positive PC were significantly associated with increased recurrence risk, with positive cytology being the strongest predictor. Similarly, older age (≥ 60 years) and positive PC were significant factors associated with reduced OS, with positive cytology being the most strongly correlated factor.

**Table 2:** Multivariable Cox analysis with recurrence-free survival and overall survival in patients with stage IA endometrial cancer RFS: recurrence-free survival, OS: overall survival, HR: hazard ratio, CI: confidence interval, BMI: body mass index, LVSI: lymphovascular space invasion, Statistical significance of differences between groups was conducted using likelihood ratio test. A p-value < 0.05 was considered statistically significant.

	RFS		OS	
Characteristic	HR (95% CI)	P-value	HR (95% CI)	P-value
Age (years)	-	-	-	-
<60	1	-	1	-
>60	4.0 (1.62–10.8)	0.0025	5.58 (1.46–28.4)	0.01
Median BMI (kg/m^2^)	-	-	-	-
<30	1	-	1	-
>30	0.73 (0.17–2.27)	0.62	0.43 (0.02–2.42)	0.38
Final pathology	-	-	-	-
Endometrioid	1	-	1	-
Non-endometrioid	0.61 (0.09–2.44)	0.51	0.48 (0.02–3.07)	0.48
LVSI	-	-	-	-
No	1	-	1	-
Yes	2.20 (0.56–7.02)	0.24	4.0 (0.81–20.1)	0.86
Surgical procedure	-	-	-	-
Open surgery	1	-	1	-
Minimally invasive surgery	0.26 (0.08–0.70)	0.0073	0.47 (0.07–2.19)	0.35
Adjuvant therapy	-	-	-	-
No	1	-	1	-
Yes	0.89 (0.30–2.47)	0.82	3.9 (0.88–22.1)	0.07
Peritoneal cytology	-	-	-	-
Negative	1	-	1	-
Positive	8.33 (2.41–25.8)	0.0016	14.2 (2.22–90.3)	0.0067

## Discussion

In this study, we analyzed the prognostic impact of positive PC in stage IA endometrial cancer (FIGO 2008) and reassessed its clinical significance in the context of modern surgical practices. Among 325 patients, 19 (5.8%) had positive PC, which is consistent with previous reports indicating a positive PC incidence ranging from 3.3% to 13.7% in early-stage endometrial cancer [7-13].

Recurrence was observed in five of 19 patients with positive PC (26.3%), which was significantly higher than that in the negative PC group (18 of 306; 5.9%). Furthermore, the five-year RFS rate was 72.7% (14/19) in the positive PC group versus 93.7% (287/306) in the negative PC group (p = 0.0001), and the five-year OS rate was 82.1% (15/19) versus 96.1% (294/306), respectively (Figures 1, 2). These data underscore the clinical importance of Tables 1, 2, which demonstrate the disproportionate distribution of recurrence and survival based on PC status, despite similarity in most other clinicopathological characteristics.

Cox proportional hazards analysis identified positive PC as the most significant factor associated with both recurrence (HR 8.33, 95% CI: 2.41-25.8) and OS (HR 14.2, 95% CI: 2.22-90.3). These findings are consistent with previous large-scale studies. For example, Seagle et al. [8] reported a four-year OS rate of 79.5% in patients with positive PC versus 92.2% in those with negative PC in stage I/II disease and identified positive PC as an independent prognostic factor (HR 1.85, 95% CI: 1.28-2.67). Similarly, Matsuo et al. [7] demonstrated an association between positive PC and decreased cause-specific survival even in low-risk endometrial cancer (HR 1.64, 95% CI: 1.01-2.68). However, the extent to which MIS was incorporated in those studies remains unclear.

In our cohort, 179 patients (55.1%) underwent MIS, reflecting current clinical practice. To reduce bias associated with surgical manipulation, no uterine manipulators were used, and the external cervical os was sutured prior to surgery to prevent tumor spillage into the peritoneal cavity. This procedural consistency allowed for a clearer evaluation of the true prognostic significance of positive PC, independent of iatrogenic dissemination.

Siegenthaler et al. [14] previously reported that patients with positive PC were more likely to experience intra-abdominal or distant recurrence, as opposed to local recurrence. In our study, of the five recurrences in the positive PC group, four involved peritoneal or distant metastases, and only one involved local recurrence. These recurrence patterns highlight the need for vigilant surveillance strategies targeting intra-abdominal and distant sites in patients with positive PC.

Additionally, it has been suggested that surgical instruments like uterine manipulators may facilitate tumor cell dissemination. The Laparoscopic Approach to Cervical Cancer (LACC) trial [15] indicated poorer oncologic outcomes in the MIS group, potentially due to peritoneal spread via manipulators. While MIS is now an established standard of care for early-stage endometrial cancer [16,17], few trials have examined the specific oncologic impact of manipulator use. A recent prospective study [14] observed cytologic conversion from negative to positive in 10 of 124 cases after manipulator use, suggesting a causal link. Our study, devoid of manipulator-related variables, supports the notion that positive PC is an independent adverse prognostic factor.

Regarding adjuvant therapy, six of 19 patients (31.6%) in the positive PC group received postoperative treatment, compared to 57 of 306 (18.6%) in the negative PC group (p = 0.17). Notably, two recurrences occurred in patients with positive PC who had no additional risk factors and did not receive adjuvant therapy. While the small number of positive PC cases limited our ability to evaluate the efficacy of adjuvant chemotherapy statistically, our findings align with previous reports suggesting that postoperative chemotherapy may improve survival outcomes in this population [8,18].

The strength of our study lies in the targeted analysis of positive PC in stage IA disease within a standardized surgical context, including contemporary MIS protocols without uterine manipulators. However, a key limitation is the relatively small number of positive PC cases, which may affect the statistical power and generalizability of the results. Furthermore, as a retrospective study based on medical record data, inherent biases cannot be excluded.

## Conclusions

Our study demonstrated that positive PC is associated with significantly increased recurrence and reduced survival in patients with stage IA endometrial cancer. These findings were independent of surgical approach and highlight the importance of considering positive PC as a key prognostic factor. Given the risk of peritoneal or distant recurrence in these patients, careful postoperative monitoring and individualized consideration of adjuvant therapy may be warranted. These results support the reevaluation of current clinical strategies and underscore the need for prospective studies to clarify the role of PC in treatment decision-making for early-stage endometrial cancer.

## References

[1] Colombo N, Creutzberg C, Amant F (2016). ESMO-ESGO-ESTRO consensus conference on endometrial cancer: diagnosis, treatment and follow-up. Ann Oncol.

[2] Concin N, Matias-Guiu X, Vergote I (2021). ESGO/ESTRO/ESP guidelines for the management of patients with endometrial carcinoma. Int J Gynecol Cancer.

[3] Abu-Rustum N, Yashar C, Arend R (2023). Uterine neoplasms, version 1.2023, NCCN Clinical Practice Guidelines in Oncology. J Natl Compr Canc Netw.

[4] Pecorelli S (2009). Revised FIGO staging for carcinoma of the vulva, cervix, and endometrium. Int J Gynaecol Obstet.

[5] Kasamatsu T, Onda T, Katsumata N (2003). Prognostic significance of positive peritoneal cytology in endometrial carcinoma confined to the uterus. Br J Cancer.

[6] Tebeu PM, Popowski Y, Verkooijen HM, Bouchardy C, Ludicke F, Usel M, Major AL (2004). Positive peritoneal cytology in early-stage endometrial cancer does not influence prognosis. Br J Cancer.

[7] Matsuo K, Matsuzaki S, Nusbaum DJ (2020). Malignant peritoneal cytology and decreased survival of women with stage I endometrioid endometrial cancer. Eur J Cancer.

[8] Seagle BL, Alexander AL, Lantsman T, Shahabi S (2018). Prognosis and treatment of positive peritoneal cytology in early endometrial cancer: matched cohort analyses from the National Cancer Database. Am J Obstet Gynecol.

[9] Takenaka M, Kamii M, Iida Y (2021). Re-thinking the prognostic significance of positive peritoneal cytology in endometrial cancer. Gynecol Oncol.

[10] Garg G, Gao F, Wright JD, Hagemann AR, Mutch DG, Powell MA (2013). Positive peritoneal cytology is an independent risk-factor in early stage endometrial cancer. Gynecol Oncol.

[11] Scott SA, van der Zanden C, Cai E, McGahan CE, Kwon JS (2017). Prognostic significance of peritoneal cytology in low-intermediate risk endometrial cancer. Gynecol Oncol.

[12] Shiozaki T, Tabata T, Yamada T, Yamamoto Y, Yamawaki T, Ikeda T (2014). Does positive peritoneal cytology not affect the prognosis for stage I uterine endometrial cancer?: the remaining controversy and review of the literature. Int J Gynecol Cancer.

[13] Kanno M, Yunokawa M, Nakabayashi M (2022). Prognosis and adjuvant chemotherapy for patients with positive peritoneal cytology in stage IA endometrial cancer. Sci Rep.

[14] Siegenthaler F, Johann S, Imboden S (2022). Prospective multicenter trial assessing the impact of positive peritoneal cytology conversion on oncological outcome in patients with endometrial cancer undergoing minimally invasive surgery with the use of an intrauterine manipulator: positive peritoneal cytology conversion and its association with oncological outcome in endometrial cancer. Ann Surg Oncol.

[15] Ramirez PT, Frumovitz M, Pareja R (2018). Minimally invasive versus abdominal radical hysterectomy for cervical cancer. N Engl J Med.

[16] Walker JL, Piedmonte MR, Spirtos NM (2012). Recurrence and survival after random assignment to laparoscopy versus laparotomy for comprehensive surgical staging of uterine cancer: Gynecologic Oncology Group LAP2 study. J Clin Oncol.

[17] Janda M, Gebski V, Davies LC (2017). Effect of total laparoscopic hysterectomy vs total abdominal hysterectomy on disease-free survival among women with stage I endometrial cancer: a randomized clinical trial. JAMA.

[18] Matsuo K, Yabuno A, Hom MS (2018). Significance of abnormal peritoneal cytology on survival of women with stage I-II endometrioid endometrial cancer. Gynecol Oncol.

